# Frequency and correlates of suicidality in a sample of traumatized treatment-seeking refugees

**DOI:** 10.1186/s12888-025-07703-5

**Published:** 2026-01-24

**Authors:** Mirjam Sophie Rueger, Kai Jannik Nehler, Franziska Lechner-Meichsner, Thomas Ehring, Mia Maria Günak, Nexhmedin Morina, Sascha Kuck, Ricarda Mewes, Julia Giesebrecht, Cornelia Weise, Regina Steil

**Affiliations:** 1 Department of Psychology, Goethe University Frankfurt, Varrentrappstr. 40-42, 60486 Frankfurt, Germany; 2 Department of Psychology, HMU Health and Medical University Erfurt, Dalbergsweg 5, 99084 Erfurt, Germany; 3 Department of Psychology, University of Wuppertal, Klotzbahn 3, 42105 Wuppertal, Germany; 4 Department of Psychology, Utrecht University, Heidelberglaan 1, Utrecht, 3584 CS the Netherlands; 5 Department of Psychology, LMU Munich, Leopoldstr. 13, 80802 Munich, Germany; 6 German Center for Mental Health (DZPG), Munich-Augsburg site, Munich, Germany; 7 Institute of Psychology, University of Münster, Münster, Germany; 8 Institute of Psychology, University Klagenfurt, Universitätsstraße 65-67, Klagenfurt a.W, 9020 Austria; 9 Department of Psychology, Philipps-University of Marburg, Gutenbergstr. 18, Marburg, 35037 Germany; 10 Department of Psychology, Clinical Psychology and Behavioral Health Technology, Friedrich-Alexander-Universität Erlangen-Nürnberg, Nägelsbachstr. 49b, 91052 Erlangen, Germany

**Keywords:** Suicidality, cPTSD, PTSD, Depression, Correlates, Treatment-seeking refugees, Insecure asylum status

## Abstract

**Introduction:**

Refugees are at heightened risk for suicidality due to trauma exposure, elevated prevalence of mental disorders, and postmigration stressors. Therefore, it is important to study Suicidal Behavior Disorder (SBD), included in DSM-5 for research purposes, as well as correlates of suicidality in this population.

**Methods:**

In a sample of *N* = 103 treatment-seeking refugees, SBD frequency was assessed using a clinical interview. Associations between suicidality (presence and severity) and potential correlates - gender, age, Posttraumatic Stress Disorder (PTSD), Disturbances in Self-Organization (DSO), depression, insecure asylum status, flight duration, postmigration living difficulties (PMLD) and religiosity - were examined via univariate and multivariate analyses.

**Results:**

Fifteen patients (14.66%) met criteria for SBD. DSO were associated with the presence of suicidality in univariate and multivariate analyses and with the severity of suicidality in multivariate analyses. Sexual trauma frequency was associated with the presence of suicidality, while PTSD severity and lower religiosity were linked to greater suicidality severity. Depressive symptoms were associated with suicidality only in univariate analyses while an insecure asylum status was associated with suicidality in multivariate analyses only.

**Conclusion:**

Suicidality should be systematically assessed in treatment-seeking refugees, particularly those with DSO or an insecure asylum status. Brief suicide prevention interventions may be warranted when treatment duration is uncertain, and targeted PTSD treatments should be offered when indicated.

**Clinical trial registration:**

The RCT was pre-registered at the German Clinical Trials Register (DRKS 00019876) on 28th of April 2020. The specific research question and analyses of this investigation were not pre-registered separately.

## Introduction

According to the dictionary of the American Psychological Association (APA) suicidality can be defined as the suicide risk, which is normally evident through suicidal ideation, intent or plan [1]. The Diagnostic and Statistical Manual of Mental Disorders (DSM) added the new diagnosis of Suicidal Behavior Disorder (SBD) to their 5th edition, which explicitly requires a suicide attempt within the last 24 months for diagnosis, rather than suicidal ideation or preparatory acts alone [2]. To make a diagnosis of SBD, clinicians must further determine the intent to die, ensuring that the harm did not occur in the context of non-suicidal self-injury, delirium, confusion or for political or religious reasons. It was included in the diagnostic system to acknowledge that suicidal behavior is not limited to depression or borderline personality disorder, which are the only disorders currently including a symptom of suicidal behavior [3]. Therefore, this inclusion aims to facilitate the identification of suicidal behavior in diagnostic assessments, ensuring that the diagnosis adheres to established validity and reliability criteria for mental disorders [3]. There are numerous psychological theories aiming to explain suicidality and specifically the distinction between suicidal ideation and suicide attempts [4]. The interpersonal theory of suicide posits that suicidal ideation develops when an individual experiences thwarted belongingness and perceived burdensomeness, while suicides or suicide attempts occur if an individual aquires suicidal capability [5, 6]. Following this theory, refugees might be at a heightened risk for both suicidal ideation and behavior. Thwarted belongingness encompasses factors such as social isolation, loneliness, and the absence of reciprocal caring relationships, which are often present in refugees through postmigration living difficulties [7–9]. In line with this, studies have shown that refugees with suicidal ideation are less likely to be partnered [10, 11], or have information about their family members left behind [10]. Similarly, they are more likely to be kept in detention [12, 13], and experience higher levels of social isolation [9].

Perceived burdensomeness includes feelings of being a liability and self-hate. This factor can be assumed to be present in refugees because they often experience visa insecurity, which might make them feel unwanted and has been shown to be associated with suicidal thoughts and intent [11, 14]. Additionally, many refugees are not allowed to work during the asylum process or might experience difficulties in securing employment afterwards, which might also contribute to feelings of being a burden. This is important since the employment status has been shown to be associated with suicidal ideation [7, 15]. Furthermore, most refugees experience traumatic events in their home countries or during flight [16], which is associated with high prevalence of related disorders like Posttraumatic Stress Disorder (PTSD) or depression. These disorders often include feeling worthless or altered self-cognitions in general [2, 17]. Those feelings can align with perceived (self-) burdensomenss, which has been shown to predict suicidal ideation [18]. Generally, mental disorders are highly associated with suicidality [19–21] and around 90% of people who commit suicide have previously been diagnosed with a mental disorder [22, 23]. It is also hypothesized that traumatic experiences might lead to acquired suicidal capability, because exposure to physically painful and fearful aspects might reduce fear of harming oneself [6]. Religiosity has been identified as a protective factor [8], consistent with the interpersonal theory of suicide, because religious individuals may experience an enhanced sense of belonging. Additionally, specific religious norms may prohibit suicidal behaviors [8].

In line with the argumentation above, the World Health Organization (WHO) states that experiencing conflict, disaster, violance, abuse, but also discrimination puts people at a heightened risk of suicide [24], which means that refugees are particularly at risk. Indeed, high prevalences of suicidality in refugees have been reported. An umbrella meta-analysis found significantly higher suicide death rates, suicide attempt rates, suicide plan rates and suicidal ideation in refugees as compared to residents [25]. Another meta-analysis found that the prevalence of suicidality is five times higher in refugees than regular immigrants [26]. Generally, studies found rates of suicidal ideation in refugees between 7.5% and 46% [9, 10, 15, 25–28] as compared to a pooled point prevalence of 3.96% in the European general population [29]. Haase and colleagues [27] concluded that refugees are at a high suicide risk before, during, and after flight. A diagnosis of PTSD and PTSD severity are associated with suicidality in general and in refugees, specifically [7, 10, 14, 30–32], even though depression has been suggested as a mediator for these relationships [32]. Generally, PTSD prevalence in refugees is higher than in the general population (~ 30% in refugees as compared to ~ 4–5% in the general population; [33, 34]). The 11th edition of the International Classification of Diseases (ICD-11) has added complex PTSD (cPTSD) as a sibling disorder to PTSD [17] that is characterized by Disturbances in Self-Organization (DSO), i.e., affect dysregulation, a negative self-concept and difficulties in relationships, in addition to the core PTSD symptoms of re-experiencing, avoidance, and hyperarousal. It has been hypothesized that cPTSD might possibly have even higher associations with suicidality than PTSD alone due to greater trauma exposure, more complex symptom presentations in adulthood, greater functional impairment and less well-being [35]. People with prolonged and severe trauma exposure are more likely to develop cPTSD and therefore a high probable prevalence has been found in refugees [36]. One study found that cPTSD but not PTSD is related to suicidality [35], while another study found a higher suicide risk in a cPTSD as compared to a PTSD group [37]. To the best of our knowledge, there is no publication to date on the association of cPTSD and suicidality in refugees. Gelezelyte and colleagues [38] hypothesized that cPTSD may mediate the relation between sexual abuse and suicide risk in general. This is relevant given that in refugees who recently arrived in Germany sexual violence specifically predicted suicidality, while all other trauma types did not [10]. Other significant predictors in that study were younger age, longer flight duration, PTSD and depression severity [10].

One other factor that is often associated with suicidality is gender [39]. It is often assumed that women engage in suicidal behaviors more often but men are more likely to complete a suicide [40]. In refugees, one meta-analysis found more suicidal ideation in men [25] whereas another found the opposite [26]. Additionally, female gender is associated with some of the risk factors for suicidality like depression and PTSD [41] that are often present in refugees, which makes gender an important factor to consider in relation to suicidality in refugees.

Taken together, the prevalence of suicidality is high in refugees, which can be explained by risk factors such as high trauma exposure, visa insecurity, or PTSD (e.g., 10, 11). Generally, research on suicidality is difficult given the relatively low base rate and therefore the need for large samples [6]. Given the additional difficulties in recruiting refugees for studies, research on suicidality in refugees is particularly limited. Estimating the frequency of SBD in refugees might help to determine whether the proposed DSM-5 diagnosis has incremental validity across diverse cultural and trauma contexts. It might further inform targeted suicide prevention and treatment efforts. Therefore, the present study aimed to assess the frequency of SBD and evaluate correlates for suicidality (i.e., gender, age, flight duration, postmigration living difficulties, visa security, severity of PTSD, DSO, and depression as well as religiosity) in a sample of treatment-seeking traumatized refugees. This is important for evaluating and contextualizing suicide risk during an assessment, as well as for informing treatment approaches for refugees more broadly.

## Materials and methods

### Participants and procedure

We used the baseline data of a multi-center randomized controlled trial (RCT), which investigated the efficacy of imagery rescripting as a brief intervention for adult refugees with PTSD as compared to usual care and treatment advice [42]. The RCT was pre-registered at the German Clinical Trials Register (DRKS 00019876, registered on 28th of April 2020), while the specific research question and data analysis of this investigation were not pre-registered separately. Participants were recruited between 2019 and 2024 at four university outpatient treatment facilties in different German cities. The trial was carried out according to the Declaration of Helsinki, approved by the ethics committee of the German Psychological Association (SteilRegina2019-10-18-VA, SteilRegina2020-02-26-AM) and all participants provided written informed consent. During the baseline assessments clinical interviews were conducted by trained clinician raters either with or without the help of a trained interpreter and participants completed self-report questionnaires, which were available in English, German, Arabic, and Farsi. If participants were not fluent in any of those languages or if they were illiterate, the interpreter helped them to complete the self-report instruments. All participants who had completed the clinical interviews were included in the current study (*N* = 103).

### Measures

#### Suicidality and depression severity

The structured Mini Neuropsychiatric Interview (MINI; [43]) was used to assess SBD, suicidality- and depression-severity. The interview has demonstrated good psychometric properties [44, 45] and was developed to be brief but accurate in the diagnosis of DSM-5 and ICD-10 disorders. According to the MINI, a severity score can be calculated for suicidality by summing the points each item was assigned by Sheehan [43] in case it is answered affirmatively (see Table 2) and can range between 0 and 169. Suicidality can be categorized according to the suicidality points as none (0 points), low (1–8 points), moderate (9–16 points) and high (≥ 17 points). In the depression part of the MINI, patients are asked about each symptom of depression according to ICD-10 and depending on their answers to the typical symptoms, the accessory symptoms can be skipped. They are asked for both current as well as previous depressive symptoms using a binary format (yes vs. no). For depression severity, a sum score was calculated by assigning a 1 to all affirmative answers of current depressive symptoms. This sum score can thus range between 0 and 9.

#### PTSD-ICD-11- and DSO-severity

To assess PTSD-ICD-11- and DSO-severity, the Complex PTSD Item Set additional to the CAPS was used (COPISAC; [46]). Regarding PTSD-ICD-11-severity, the core symptoms were assessed with six matched items from the Clinican Administered PTSD Scale for DSM-5 (CAPS-5; [47]) and summed to provide a total score. This score can range between 0 and 24, because the items can be rated between 0 (*absent*) and 4 (*extreme*). For DSO-severity, the four additional items from COPISAC (i.e., one item each for affect regulation and self-concept and two items for difficulties in relationships) were summed to retain a total score, which ranges between 0 and 16. Internal consistencies in our sample were acceptable with Mc Donald’s Omega = 0.72 for PTSD-ICD-11-items and 0.79 for DSO-items, respectively.

#### Sexual trauma history

The Life Events Checklist for the Diagnostic and Statistical Manual of Mental Disorders – Interview Version (LEC-5; [48]) was used in the extended version by Lechner-Meichsner and Steil [46] to assess the sexual trauma history. Patients were also asked how often they had been exposed to each event (i.e., sexual assault, other unwanted or uncomfortable sexual experience, repeated childhood sexual abuse), and their answers were rated as 1 (*one time*), 2 (*2–3 times*), 3 (*4–10 times*) and 4 (*uncountable times*) if the event met criterion A [49]. Then, a frequency score was calculated as a sum of these ratings.

#### Postmigration living difficulties

To assess post-migration stressors, the 27-item Postmigration Living Difficulties Questionnaire (PMLD; [50]) was used in an adapted version [51]. Patients rated if any of the items (e.g., discrimination) had been a problem over the previous year on a 5-point Likert response format from 0 (*No problem at all*) to 4 (*A very serious problem*). A composite score was used for our analyses, which can range between 0 and 108. The PMLD showed acceptable internal consistency in the present sample with Mc Donald’s Omega = 0.89.

#### Sociodemographic variables

Sociodemographic varibles including gender, age, flight duration, security of asylum status and religiosity were assessed in a structured interview and administered by trained clinician raters. A temporary residence permit and a tolerance permit were categorized as 0 (*insecure asylum status*) while a permanent residence permit, a citizenship and a settlement permit were categorized as 1 (*secure asylum status*). Religiosity was assessed with a 5-point Likert response format as 1 (*I am a religious believer and visit the church/ mosque to fulfill my religious obligations*), 2 (*I am a religious believer and fulfill my religious obligations as an individual*), 3 (*I believe in God but I am not religious*), 4 (*I am spiritual but not religious*) and 5 (*I am not religiously influenced*).

### Data analysis

All analyses were conducted in R version 4.5.1 [52]. Descriptive statistics were used to evaluate sample characteristics. There were between 1.3% and 11.7% missing values per measure. Multiple imputation was performed for missing values using fully conditional specification with a maximum of ten iterations via the package mice version 3.18.0 [53]. During the imputation process, post-processing rules were applied to remove impossible combinations of item values. All missing data were imputed 20 times, resulting in 20 complete datasets. The following analyses were conducted separately for each imputed dataset and pooled according to Rubin’s rules [54], with the exception of parts of the two-part regression model, as described below.

The internal consistency was calculated using Mc Donald’s Omega with the package psych version 2.5.6 [55]. It was then evaluated how many patients fulfilled the diagnosis of SBD by assessing the number and percentage of patients who tried to commit suicide within the last 24 months. The other MINI suicidality items (e.g., suicidal ideation, suicide plan) were also evaluated descriptively. The severity scores of suicidality and depression as well as PTSD-ICD-11-, and DSO, and the PMLD composite score were then calculated.

Given the challenges inherent in researching suicidality in refugees, a two-part approach was adopted by first examining correlates associated with reporting any suicidality compared to none. Then, it was investigated how these correlates are associated with the severity of suicidality, summarizing both suicidal ideation and suicidal behaviors in one score because of the low base rate of suicidal behavior. Bivariate relationships were explored between suicidality and a range of potential correlates (i.e., depression severity, PTSD-ICD-11 severity, DSO severity, frequency of sexual trauma, PMLD score, gender, age, flight duration, and insecure asylum status, religiosity). As previously described, suicidality can be conceptualized as occurring in distinct stages: no suicidality, represented by a score of zero, and suicidal thoughts and/ or behavior, represented by a score greater than zero on the interview. To reflect this distinction, two sets of associations were calculated. First, suicidality was dichotomized (0 = no suicidality, 1 = suicidal thoughts or behavior), and it was examined how the predictors were associated with the likelihood of belonging to the suicidal category. Second, the sample was restricted to individuals with a suicidality score greater than zero and it was investigated how the same predictors were related to the severity of suicidality within this subgroup. Associations were assessed using zero-order Pearson correlations for pairs of continuous or dichotomous variables, and point-biserial correlations for associations between dichotomous and continuous variables.

Expanding to a multivariate framework, it was explored which correlates were associated with suicidality while controlling for one another, using a two-part regression model implemented with the twopartm package (version 0.1.0; [56]). The first part of the model predicted the presence versus absence of suicidality, while the second part examined the associations between predictors and the severity of suicidality among individuals with nonzero suicidality scores. This structure mirrors the one used in the bivariate analyses described earlier. For both parts of the model, exploratory identification of influential predictors was performed using a stepwise procedure based on the Bayesian Information Criterion (BIC). Rather than conducting model selection separately within each imputed dataset, which could result in differing sets of predictors across imputations, a stacked approach was applied by combining the 20 imputed datasets into one and adjusting for the artificially increased sample size in the computation of the BIC. This selection strategy, based on stacked data, follows procedures that have been validated in the context of multiple regression within network analysis [57]. After model selection, explained variance was estimated separately for both model parts. Logistic regression was used for the first part and Gamma regression with a log link for the second, each fitted within every imputed dataset. Model fit was quantified using pseudo R² measures from the performance package (version 0.15.2; [58]), applying Tjur’s [59] definition for the logistic model and Nagelkerke’s [60] approach for the Gamma model. Following van Ginkel [61], the resulting values were averaged across imputations to obtain descriptive pooled estimates of model fit for both parts.

## Results

### Sample characteristics

Data from *N* = 103 participants were included in the current study (see Table 1). They were mostly male (64.07%, *n* = 66) and their age ranged between 18 and 62 years (*M* = 32.88, *SD* = 11.36). Fourty-five participants (43.68%) had a secure asylum status and the mean flight duration was 13.34 months (*SD* = 33.62). The mean PMLD composite score was *M* = 43.16 (*SD* = 19.00). The mean symptom severities were *M* = 6.18 (*SD* = 3.00) for depression, *M* = 11.76 (*SD* = 4.30) for PTSD-ICD-11, and *M* = 5.59 (*SD* = 3.97) for DSO. Twenty-four participants (23.30%) reported that they were religious and go to a church, mosque or similar to fulfill their religious obligations, while 44 participants (42.71%) reported that they were religious but fulfill their religious obligations as an individual. Fifteen participants (14.56%) reported that they believe in God but were not religious, two participants (1.94%) said they were spiritual but not religious and 14 participants (13.59%) reported not being religiously influenced.

**Table 1:** Demographic and clinical characteristics of the sample (N = 103)

Variable	*n*	%	M	SD
Gender^a^ Male Female Other	66 33 3	64.07% 32.03% 2.91%		
Age (years)^b^			32.88	11.36
Country of origin^c^ Afghanistan Syria Iraq, Iran Nigeria Sierra Leone Eritrea, Guinea, former Yugoslavia, Pakistan, Palestine, Turkey Cameroon, Egypt, Jordan, Paraguay, stateless, Tajik, Tanzania	34 22 8 each 5 4 2 each 1 each	33.01% 21.35% 7.76% each 4.85% 3.88% 1.94% each 0.97% each		
Religion^c^ Muslim Christian Non-denominational Other	73 15 10 2	70.87% 14.56% 9.71% 1.94%		
Marital Status^b^ Single, living with family Single, living alone Married Divorced Separated Widowed In Partnership	11 43 23 8 4 2 10	10.67% 41.74% 22.33% 7.76% 3.88% 1.94% 9.71%		
Residence status^b^ Permanent Residence Permit Temporary Residence Permit Tolerance Permit Other	39 34 22 6	37.86% 33.01% 21.35% 5.82%		
Time since arrival in Germany (years)^b^			5.25	4.63
Formal education (years)^c^			8.94	4.3
Housing situation^b^ Own apartment Living with friends/family Community housing facility Initial reception facility Other	40 3 47 4 7	38.83% 2.91% 45.63% 3.88% 6.79%		
Currently employed^b^	34	33.01%		

Note. LEC-5 = Life Event Checklist in the adapted version by Lechner-Meichsner & Steil [46]; CAPS = Clinician-Administered PTSD Scale for DSM-5; COPISAC = Complex PTSD Item Set additional to the CAPS; ITQ = International Trauma Questionnaire; GHQ-28 = General Health Questionnaire; ADES-8 = Adolescent Dissociative Experiences Scale; PSQI = Pittsburgh Sleep Quality Index. Patients had missing values on some measures and we used pairwise exclusion for all analyses

^a^ *n* = 102

^b^ *n* = 101

^c^ *n* = 100

### Prevalence and severity of suicidality

The pooled relative frequency of SBD was 14.66% (*n* = 15^1^). Twenty-five participants^1^ (24.17%) reported no suicidality, while 31.50% (*n* = 33^1^) of participants were classified as having low, 8.93% (*n* = 9^1^) as having moderate and 35.38% (*n* = 36^1^) as having high suicidality. Frequencies of the single MINI-items (e.g., suicidal ideation, suicide plan) are depicted in Table 2. The mean of the suicidality points was *M* = 15.00 (*SD* = 20.07), indicating moderate suicidality.

**Table 2:** Suicidality items according to mini neuropsychiatric interview (MINI)

MINI- sucicidality item	Points if affirmed	*n* (%)^1^
Thinking about being better off dead/ wishing one was dead/ thinking about needing to be dead	1	62 (60.24)
Thinking about harming/ hurting/ injuring oneself with some intent that one might die/ thinking about suicide	6	33 (31.75)
Hearing voice telling one to kill oneself/ dreams with suicidal content	4	13 (13.06)
Suicide method in mind	8	23 (22.62)
Suicide means in mind	8	11 (10.87)
Place to attempt suicide in mind	8	13 (12.82)
Date/ timeframe to attempt suicide in mind	8	5 (4.85)
Thinking about tasks to complete before attempting suicide	8	7 (6.80)
Intention to act on suicidal thoughts	8	4 (4.03)
Intention to die as a result of suicidal act	8	6 (6.06)
Need/ impulse to kill oneself/ plan to kill oneself sooner rather than later	8	6 (5.92)
Difficulties resisting suicidal impulses	8	3 (2.91)
Active steps to prepare for suicide attempt a) Prepared, but did not start the attempt b) Prepared, but aborted c) Prepared, but were interrupted	9 10 11	6 (6.02) 2 (1.94) 2 (1.94) 1 (0.97)
Injured oneself without intention to kill oneself	0	10 (10.05)
Suicide attempts in the past month a) Start, but then decided to stop b) Start, but were interrupted c) Went through with the attempt as planned	12 13 14	2 (2.04) 2 (1.94) 0 (0.00) 0 (0.00)
Suicide attempts (lifetime)	4	41 (39.32)

Note. All items in the MINI refer to the last month except for lifetime suicide attempts. There is one further item, which gets assigned points: How likely are you to try to kill yourself within the next 3 months on a scale of 0-100%? Any percentage > 0% is counted as affirmed and gets 13 points

^1^ All ns and percentages refer to the pooled results after multiple imputation. For all ns, we rounded to integers to increase comprehensibility

### Correlates of suicidality

The Pearson and point-biserial associations of experiencing any suicidality with the correlates are depicted in Table 3. Experiencing any suicidality was positively and significantly associated with severity of depression and DSO as well as the frequency of sexual traumatic events. Therefore, the higher the depression-, and DSO-severity as well as the frequency of sexual traumatic events, the more likely it was that a patient experienced any suicidality. Pearson and point-biserial associations of suicidality severity with correlates in the subgroup of patients experiencing any suicidality are shown in Table 4. The severity of suicidality was positively and significantly associated with depression-, and PTSD-ICD-11-severity. It was also positively and significantly associated with religiosity, thus the suicidality was more severe in patients that reported lower religiosity since the highest religiosity value of 5 stated “*I am not religiously influenced”.*

**Table 3:** Pearson and point-biserial correlations of the binary variable of suicidality with potential correlates

	1.	2.	3.	4.	5.	6.	7.	8.	9.	10.	11.
1. Suicidality (no vs. yes)	-										
2. Gender	0.14										
3. Age	0.08	0.27**									
4. Depression severity	0.23*	− 0.03	− 0.03								
5. PTSD-ICD-11-severity	0.14	0.08	0.17	0.32*							
6. DSO-severity	0.23*	0.09	0.17	0.21*	0.33***						
7. Frequency of Sexual traumatic events	0.21*	0.23*	0.06	0.23*	0.22*	0.29**					
8. Secure asylum status	− 0.20	0.11	0.16	− 0.20*	− 0.08	0.23*	− 0.03				
9. Flight duration	− 0.02	− 0.01	0.09	0.04	0.04	− 0.00	0.05	− 0.16			
10. Sum PMLD	0.17	0.12	− 0.04	0.16	0.14	0.04	0.01	− 0.29**	0.06		
11. Religiosity^1^	− 0.10	− 0.26**	0.01	− 0.04	− 0.01	− 0.10	0.18	0.06	− 0.11	− 0.33***	

Note. PTSD-ICD-11 = Posttraumatic stress disorder according to ICD-11, DSO =  disturbances in self-organization, PMLD = postmigration-living difficulties

No Suicidality was depicted as 0 while any suicidality as depicted as 1. All correlations and *p*-values refer to pooled results after multiple imputation

* *p* < .05, ** *p* < .01, *** *p* < .001

^1^ Religiosity was assessed with a 5-point Likert response format as 1 (*I am a religious believer and visit the church/ mosque to fulfill my religious obligations*), 2 (*I am a religious believer and fulfill my religious obligations as an individual*), 3 (*I believe in God but I am not religious*), 4 (*I am spiritual but not religious*) and 5 (*I am not religiously influenced*)

**Table 4:** Pearson and point-biserial correlations of suicidality severity with potential correlates in the subgroup of participants with any suicidality

	Gender	Age	Depression severity	PTSD-ICD-11-severity	DSO-severity	Frequency of sexual traumatic events	Secure asylum status	Flight duration	Sum PMLD	Religiosity^1^
Suicidality	− 0.18	− 0.01	0.25*	0.36**	0.15	0.13	− 0.22	0.04	0.11	0.30**

Note. PTSD-ICD-11 = Posttraumatic stress disorder according to ICD-11, DSO =  disturbances in self-organization, PMLD = postmigration-living difficulties

All correlations and *p*-values refer to pooled results after multiple imputation

^1^ Religiosity was assessed with a 5-point Likert response format as 1 (*I am a religious believer and visit the church/ mosque to fulfill my religious obligations*), 2 (*I am a religious believer and fulfill my religious obligations as an individual*), 3 (*I believe in God but I am not religious*), 4 (*I am spiritual but not religious*) and 5 (*I am not religiously influenced*)

In our exploratory two-part multiple regression model, DSO-severity (*β* = 0.17), a secure asylum status (*β* = −1.24) and the frequency of sexual traumatic events (*β* = 0.21) were selected as predictors for the presence of suicidality in the first part. The pooled R² for the first part of the model was 0.15. In the second part, DSO severity (*β* = 0.05), religiosity (*β* = 0.28), a secure asylum status (*β* = −0.89), and the PTSD-ICD-11-severity (*β* = 0.09) were selected as predictors for the severity of suicidality. The pooled R² for the second part of the model was 0.43.

## Discussion

The aim of the present study was to assess the frequency of SBD and to examine the relationship of various correlates and the presence and severity of suicidality in a sample of treatment-seeking traumatized refugees. The frequency of suicide attempts in the current sample was higher than the one reported by Bevione et al. [25] in a meta-review of systematic reviews on refugee samples in general (~ 1% vs. 39% in our sample). However, when considering suicide attempts in the past month, frequencies were similar. The frequency of suicidal ideation was also higher than the ones reported in systematic reviews and meta-analyses (32% in our sample vs. 16%: ,26,20.5%: [27]). Again, the other authors did not investigate treatment-seeking samples and reported high heterogeneity in the publications used for their meta-analyses. The frequency of suicidal ideation was similar to the ones reported by Akinyemi et al. [15] and Nesterko et al. [10], where the latter also investigated a treatment-seeking sample of refugees recently arrived in Germany.

PTSD is known to be a risk factor for suicidality in the general population [30–32], and in refugees specifically [7, 10], and cPTSD has been suggested to be a risk factor for suicidality [35, 37]. In our analyses, DSO were significantly associated with the presence of suicidality, while PTSD was significantly associated with the severity of suicidality in univariate analyses. In multivariate analyses, DSO were a relevant predictor for both presence and severity of suicidality, while PTSD was only identified as a relevant predictor for severity of suicidality. This result implies that DSO are a correlate differentiating individuals with any suicidality from individuals without any suicidality, while the severity of PTSD might play a role for the extent of suicidality in suicidal individuals. Emotion regulation difficulties in DSO might be one reason for this association, because maladaptive emotion regulation has been associated with suicidality [62, 63]. However, suicidal ideation could in itself also be an emotion regulation strategy, which, in turn, could be reflected in emotion regulation difficulties that are part of DSO. Mental images of injury or death have been found to also lead to positive emotions and have been termed ID-images [64]. These ID-images have been found to be associated with depressive symptomatology [65]. Furthermore, they were more vivid in adolescents with PTSD after childhood abuse than in a sample of matched adolescents without any disorders and and without any childhood abuse [66]. One potential underlying mechanism could be that feelings of helplessness or entrapment foster suicidal ideation as a way of regaining a sense of control over one’s suffering, even if only in the form of imagining an ultimate escape from it [65]. It is therefore possible that such ID-images also exist in refugees and future studies need to investigate if they are linked to suicidality and psychopathology.

CPTSD was also hypothesized to mediate the relationship between sexual abuse and suicide risk [38] and Nesterko et al. [10] found sexual traumas to be a significant risk factor for suicidality when controlling for all other trauma types. In our analyses, the frequency of sexual traumatic events was associated with the presence but not the severity of suicidality in both univariate and multivariate analyses. It is thus more likely for individuals with a history of sexual traumas to experience any suicidality, however the frequency of the events itself may not be as relevant for the extent of the suicidality.

Depression is widely considered a risk factor for suicidality [7, 10, 32], which is also reflected in the inclusion of suicidality as one symptom of depression in the diagnostic systems [2, 17]. It was significantly associated with both the presence and the severity of suicidality in univariate but not multivariate analyses. Given that depression was also associated with PTSD, DSO, frequency of sexual traumatic events, and the security of the asylum status, it is possible that its unique contribution to suicidality is small in the present sample.

An insecure asylum status was associated with the presence and the severity of suicidality in multivariate analyses only. This is in line with other studies [14, 16], which identified it as a risk factor. The composite score of PMLD was not associated with suicidality, which contradicts the findings of one study [8] but is in line with another one [67]. Similarly, the flight duration was not associated with suicidality, which is different from the results of Nesterko et al. [10]. Since the insecure asylum status was associated with the PMLD composite score, it is possible that the latter was less relevant on its own or that the composite score neglects differiantial aspects of the PMLD. An insecure asylum status may be perceived as an existential threat, particularly for individuals who anticipate persecution, imprisonment, or other severe consequences if returned to their country of origin, which could explain the strong association. In contrast, other factors included in the PMLD score, such as access to resources, social contacts, or experiences of discrimination, appeared to have less additional impact in this context. Furthermore, gender and age were not related to suicidality. This is in line with some [32] but contradicts other studies [10, 19, 25]. The studies that did find associations between these variables did not find straightforward results: In Nesterko et al. [10], suicidality was related to younger age, while it was related to older age in the study by Moitra et al. [19]. Similarly, some studies found more suicidal ideation in men [14, 25, 27], whereas others found it in women [26, 31]. Generally, it is often assumed that women more often engage in suicide attempts while more men complete suicides [40]. Additionally, suicides are more differently distributed in relation to gender in high-income countries, whereas it is more equally distributed in low-income countries [39], which could explain the hetereogeneity in studies and our results given that most participants came from lower-income countries. In line with another study [8], religiosity was positively correlated with the severity of suicidality in both univariate and multivariate analyses (i.e., less religiosity was associated with more suicidality given the response format of religiosity). Our results extend the current literature, because they show that religiosity will not protect an individual from experiencing any suicidality but it might protect from suicidal behaviors due to beliefs or cultural sanctions [8]. Moreover, in many Muslim contexts suicide and even suicidal thoughts are strongly stigmatized, which was also reflected during diagnostic assessments where discussing suicidality was often experienced as difficult or accompanied by feelings of shame among participants. This stigma might partly explain why religiosity could function as a protective factor in this population.

### Strengths and limitations

A major strength of our study is that we used a comprehensive assement of different aspects of suicidality, while many previous studies only assessed suicidality with a single item (e.g., [10]). Furthermore, we applied the MINI as a clinician assessment, while in several studies suicidality was merely assessed with self-reports. Additionally, the assessment of the frequency of SBD might help inform the utility and cross-cultural applicability of this relatively new disorder. The investigation of DSO as a correlate of suicidality might help inform suicide risk evaluation and the development of new treatment approaches. Another strength is our multilayered analysis because this enables to identify more complex relationships between suicidality and its correlates.

It is a limitation that we did not employ assessments validated for culturally diverse samples, which is especially important given potential cultural differences in the acceptability of discussing suicidility [8]. Given these sanctions, many patients were also reluctant to disclose suicidality, which might have led to underreporting. Further, we only differentiated between no and any suicidality and did not implement the threefold model of the interpersonal theory that differentiates between no suicidality, suicidal desire and suicidal behaviors [6]. This was due to the low base rate of current suicidal behaviors in our study, which is a common problem in suicidality research [6]. We also used baseline data from an RCT. Our sample therefore constitutes a highly selected treatment-seeking sample, which precludes generalization of the results to all refugees or even other samples. Furthermore, collider bias is possible because of these sample characteristics [68]. Both suicidality and several correlates examined in this study - such as PTSD, DSO, or depressive symptom severity - are likely to increase the likelihood of seeking treatment. As a result of this potential selection bias, associations between these constructs might be overestimated or not fully reflect the patterns in the broader refugee population. The statistical power was generally limited in our analysis due to the small sample size. While the RCT was pre-registered, the specific research question and analyses of this investigation were not. The data were collected as part of the baseline assessment of the RCT and therefore no power calculations were conducted. In addition, correlates that were routinely assessed at baseline were selected for analysis in line with correlates and risk factors identified in previous studies.

### Implications

Suicidality is an important topic when working with refugees as a psychotherapist, a social worker, an employee in initial reception facilities, a lawyer or volunteer. This might be especially relevant when the asylum status remains insecure or following a negative asylum decision (see also [14]). At the societal level, this might underscore the need for more efficient asylum procedures to reduce the duration of visa insecurity, as well as for asylum decisions to be made with benevolence. Similarly, based on our results, political communication about desired deportation and rejection of immigration does not seem beneficial for suicidality because this may increase perceived visa insecurity.

Well-established PTSD treatments like cognitive processing therapy or prolonged exposure as well as combined treatments like dialectial behavioral therapy for PTSD (DBT-PTSD) have been shown to reduce both PTSD and suicide-related outcomes [69]. Given the associations of PTSD and DSO with suicidality, DBT-PTSD might be especially helpful since it specifically adresses emotion regulation difficulties and suicidality that can be present in DSO [70–72]. It is important to offer evidence-based treatments to patients with PTSD not only to reduce their PTSD symptoms but also to reduce suicidality in case it is present. Furthermore, brief and ultra-brief suicide prevention interventions are promising [73, 74] and could be used shortly after arrival in an insecure asylum situation if it is not foreseeable if there is enough time for PTSD treatment. An intervention of regular contact in combination with the use of safety planning cards done by community volunteers reduced suicide attempts and may thus be worthwhile to implement with relatively low effort [75]. Training support staff in a culture-sensitive way might also help identify suicidal individuals and thus prevent suicides [76] and generally, being culturally sensitive is important given the different cultural rules on talking about suicide [8]. Similarly, given that religiosity might be a protective factor for the extent of suicidality, it is important to be sensitive about religious needs and provide opportunities to practice religion.

In general, different correlates appear to be involved when determining the presence versus the severity of suicidality. This is somewhat in line with the interpersonal theory of suicidality [5, 6], which states that different factors influence the suicidal desire in contrast to factors influencing suicidal behavior.

## Conclusion

DSO, sexual traumas and an insecure asylum status emerged as important correlates in treatment-seeking refugees’ experience of suicidality. Practitioners treating refugees as well as staff and volunteers in reception facilities should be particularly mindful of suicidality. Training to identify at-risk individuals is essential, and we recommend a stepwise approach in its management. Brief suicide prevention interventions may be helpful when the available timeframe for treatment is uncertain, whereas trauma-focused- interventions could be used when a longer treatment horizon is possible, as that may reduce both PTSD -symptoms and suicidality.

## Data Availability

The participants of this study did not give written consent for their data to be shared publicly, so due to the sensitive nature of the research raw data is not available.

## Footnotes

1 For all ns of the pooled results, we rounded to integers to increase comprehensibility.

## Abbreviations

APA: American Psychological Association
CAPS-5: Clinician Administered PTSD Scale for DSM-5
COPISAC: Complex PTSD Item Set additional to the CAPS
cPTSD: Complex Posttraumatic Stress Disorder
DSM: Diagnostic and Statistical Manual of Mental Disorders
DSO: Disorders of Self-Organization
ICD: International Classification of Diseases
LEC-5: Life Events Checklist for DSM-5
MINI: Mini Neuropsychiatric Interview
PMLD: Post-Migration Living Difficulties
PTSD: Posttraumatic Stress Disorder
RCT: Randomized Controlled Trial
SBD: Suicidal Behavior Disorder
WHO: World Health Organization
